# *LINGO1* rs9652490 and rs11856808 polymorphisms are not associated with risk for multiple sclerosis

**DOI:** 10.1186/1471-2377-13-34

**Published:** 2013-04-10

**Authors:** Elena García-Martín, Oswaldo Lorenzo-Betancor, Carmen Martínez, Pau Pastor, Julián Benito-León, Jorge Millán-Pascual, Patricia Calleja, María Díaz-Sánchez, Diana Pisa, Laura Turpín-Fenoll, Hortensia Alonso-Navarro, Lucía Ayuso-Peralta, Dolores Torrecillas, Elena Lorenzo, José Francisco Plaza-Nieto, José A G Agúndez, Félix Javier Jiménez-Jiménez

**Affiliations:** 1 Department of Biochemistry and Molecular Biology, University of Extremadura, Cáceres, SPAIN; 2 Neurogenetics Laboratory, Division of Neurosciences, Center for Applied Medical Research, University of Navarra, Pamplona, SPAIN; 3 Department of Neurology, Clínica Universidad de Navarra, University of Navarra School of Medicine, Pamplona, SPAIN; 4 Department of Pharmacology, University of Extremadura, Cáceres, SPAIN; 5 CIBERNED, Centro de Investigación Biomédica en Red de Enfermedades Neurodegenerativas, Instituto de Salud Carlos III, Madrid, Spain; 6 Service of Neurology, Hospital Universitario Doce de Octubre, Madrid, SPAIN; 7 Department of Medicine, University Complutense, Madrid, SPAIN; 8 Section of Neurology, Hospital La Mancha-Centro, Alcázar de San Juan, Ciudad Real, SPAIN; 9 Centro de Biología Molecular Severo Ochoa (CSIC-UAM), Facultad de Ciencias, Universidad Autónoma, Cantoblanco, Madrid, 28049, SPAIN; 10 Section of Neurology, Hospital Universitario del Sureste, Arganda del Rey, Madrid, SPAIN; 11 Department of Medicine-Neurology, Hospital “Príncipe de Asturias”, Universidad de Alcalá, Alcalá de Henares, Madrid, SPAIN

**Keywords:** Multiple sclerosis, Genetics, Genetic polymorphisms, LINGO-1, Risk factors

## Abstract

**Background:**

Some recent experimental data suggest a possible role of LINGO-1 in the pathogenesis of multiple sclerosis (MS). In an attempt to identify genetic biomarkers related to MS susceptibility, we genotyped two common SNPs in the *LINGO1* gene which have been associated to other neurological conditions, in patients with MS and in healthy subjects. These SNPs are linked to several SNPs within the *LINGO1* gene, especially in individuals of Oriental or Caucasian descent.

**Methods:**

We analyzed the allelic and genotype frequency of two *LINGO1* variants (rs9652490 and rs11856808) in 293 patients with MS and 318 healthy controls, using KASPar assays.

**Results:**

*LINGO1* rs9652490 and rs11856808 allelic and genotype frequencies did not differ significantly between MS patients and controls. The minor allele frequencies for rs9652490 were 0.171 (95% CI = 0.140-0.201) and 0.167 (95% CI = 0.138-0.196 for cases and controls respectively (p = 0.853). For rs11856808 the minor allele frequencies were 0.317 (95% CI = 0.280-0.355) and 0.310 (95% CI = 0.274-0.346) for cases and controls, respectively (p = 0.773). Allele and genotype frequencies were unrelated with the age of onset of MS, gender, and clinical course of MS. In addition, haplotype analyses did not reveal any putative risk related to haplotypes.

**Conclusions:**

These results suggest that *LINGO1* rs9652490 and rs11856808 polymorphisms are not related with risk for MS. This study adds to other published evidence indicating that, to date, the *LINGO1* SNPs studied here could be useful risk biomarkers of developing essential tremor, but not other movement disorders.

## Background

Multiple sclerosis (MS) is a chronic inflammatory demyelinating disorder with axonal degeneration affecting the Central Nervous system, which shows three major evolutive phenotypes: relapsing-remitting, primary progressive and secondary progressive. The etiology of MS is unknown, but it is probably multifactorial, with an interplay of genetic, ethnic, geographical and environmental factors (infectious or chemical) [1-5]. It has been proposed that MS is an autoimmune disorder with susceptibility influenced, if not determined, by a relatively small number of genes [1]. Findings from studies on seasonality in MS patients’ birth, disease onset and exacerbations, as well as apparent temporal trends in incidence and gender ratio support an influential effect of viruses, metabolic and lifestyle factors on MS risk. Epstein-Barr virus, vitamin D status, and smoking are factors that may explain such epidemiological patterns [4].

A haplotype within the major histocompatibility region is the major risk factor for MS. But despite clear evidence for a genetic component additional risk, specific gene variants were not identified until the recent advent of genome-wide association studies (GWAS). Until 2010, 11 GWAS have been conducted on MS, and, together with follow-up studies, these GWAS have confirmed 16 loci with genome-wide significance [6,7]. Many of these common risk variants are located at, or near to, genes with central immunological functions (such as interleukin 2 and 7 receptors, CD58, CD6, CD40, TNFRSF1A and others) and the majority are associated with other autoimmune diseases [6,7]. A further report of the International Multiple Sclerosis Genetics Consortium identified at least 50 loci related with the risk for MS [8].

Although the underlying molecular mechanisms for the axonal degeneration are unknown, the degree of inflammatory demyelination correlates with the extent of axonal damage. This suggests an involvement of the proinflammatory mediators in inducing axonal degeneration [9]. However, the alternative possibility that axonal regeneration should be severely impaired in MS lesions could be suggested, since an accumulation of glial scar and neurite growth inhibitors provide a non-permissive environment for re-growth of damaged axons [10].

**Figure 1:**
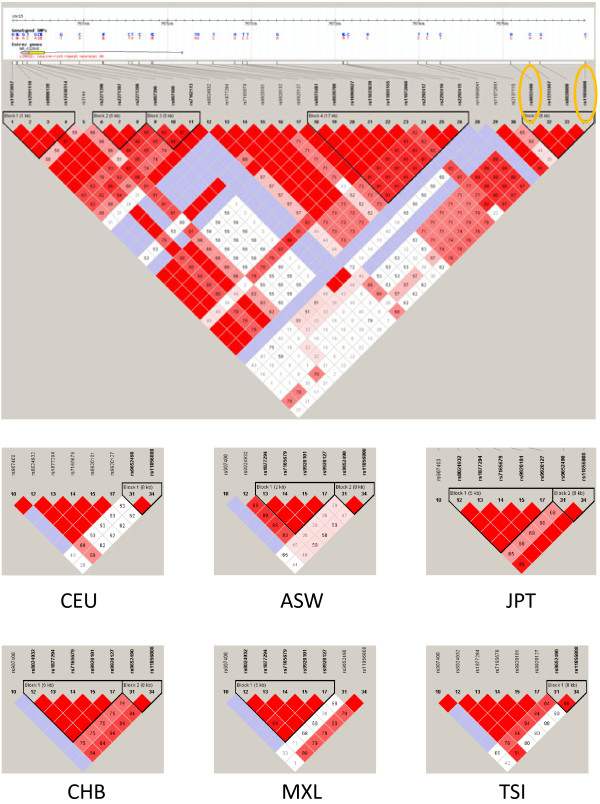
**Scheme and linkage analysis of the SNPs analyzed in this study.** The linkage figure was composed with Haploview Ver. 3, release R-2, excluding individuals with > 50% missing genotypes, according to the standard colour scheme (D’/LOD), and the D’ values (×100) are shown when relevant. Top: The area covers the whole LINGO1 gene as well as the 3’ flanking region. The SNPs tested are marked at the right side of the figure. These data correspond to Caucasian individuals (Utah residents with ancestry from northern and western Europe). Bottom: Linkage figures focusing on the two SNPs tested and six SNPs located within the LINGO1 gene. The populations correspond to: CEU, Utah residents with ancestry from northern and western Europe; ASW, African ancestry in Southwest USA; JPT, Japanese in Tokyo, Japan; CHB, Han Chinese in Beijing, China; MXL, Mexican ancestry in Los Angeles, California; TSI, Tuscany in Italia. (see the website http://www.sanger.ac.uk/resources/downloads/human/hapmap3.html).

LINGO1 (leucine rich repeat and Ig domain containing Nogo receptor interacting protein-1) has a possible role in the pathogenesis of MS. LINGO1 is a transmembrane protein expressed in neural cells which inhibits the differentiation of oligodendrocyte precursor cells into mature oligodendrocytes, as well as myelination and remyelination [11,12]. LINGO1 comprises 12 leucine rich repeats followed by an immunoglobulin (Ig) domain and a short cytoplasmic tail. It is encoded by the *LINGO1* gene (OMIM 609791, Gene Identity 84894) located in the chromosome 15q24.3 [13,14]. In neurons, LINGO1 simultaneously interacts with the Nogo-66 receptor (NgR) and p75^NTR^ or TROY to form a receptor complex that binds the structurally diverse associated glycoprotein and oligodendrocyte myelin glycoprotein, resulting in the restriction of axonal elongation via activation of the small GTPase RhoA [14-16]. Two *LINGO1* variants designated as rs9652490 and rs11856808 have been claimed to be associated in case–control GWAS with other neurological conditions such as essential tremor [17,18] and Parkinson’s disease [18,19]. Further studies confirmed the association with essential tremor, but discarded a major association with Parkinson’s disease [20-24]. These single nucleotide polymorphisms (SNPs) are, according to HapMap, tag-SNPs for the following SNPs located within the *LINGO1* gene: rs907400, rs8029432, rs1877294, rs7165679, rs9920101 and rs9920127, as well as nine additional SNPs in the 3’ flanking region of the gene. Figure 1 shows that the linkage between the two SNPs analyzed in this study and the six SNPs located within the gene differ, depending on ethnicity.

In an attempt to identify additional factors involved in MS susceptibility, we genotyped the SNPs rs9652490 and rs11856808 in the *LINGO1* gene, in patients with MS and in healthy subjects. Although *LINGO1* polymorphisms were not significantly associated with the risk and hence are not mentioned among the possible susceptibility genes in GWAS studies, the possible role of LINGO1 in the pathogenesis of MS suggests that the *LINGO1* gene should be a candidate gene for modifying MS risk.

## Methods

### Patients and controls

We recruited 293 unrelated Caucasian Spanish patients who fulfilled McDonald’s criteria for definite MS [25], with no other previous neurological diseases. Recruiting sources were the following: the “Multiple Sclerosis Association of Madrid”; n = 165 cases), the Health Areas of the Hospital La-Mancha-Centro (Alcázar de San Juan, Ciudad Real; n = 65 cases), and University Hospitals “Doce de Octubre” (Madrid, n = 32 cases), and “Príncipe de Asturias” (Alcalá de Henares, Madrid; n = 31 cases). The control group was composed of 318 healthy unrelated Caucasian Spanish individuals gender and age-matched with the patients (97 men, 221 women; mean age 43.76 ± 12.4 years). These patients participated in previous genetic studies [26-28]. The control individuals were students or professors from the University of Extremadura, Badajoz, Spain (n = 150), and the healthy spouses of patients with neurological disorders who came from different regions of Spain to the Department of Neurology, Clínica Universitaria de Navarra, University of Navarra School of Medicine, Pamplona, Spain (n = 168). All the participants were included in the study after giving written informed consent. Table 1 summarizes the characteristics of the individuals included in the study. The protocol was approved by the Ethics Committees of the University Hospitals “Príncipe de Asturias” and “Infanta Cristina” (Badajoz) and collaborating centres. The study was conducted according to the principles expressed in the declaration of Helsinki.

**Table 1:** Characteristics of the individuals included in the study

	**Overall MS patients**	**RRMS**	**SPMS**	**PPMS**	**Control individuals**
Gender (females/males)	203/90	115/44	59/32	29/14	221/97
Age (mean ± SD)	43.9 ± 11.4	40.0 ± 10.5	47.2 ± 9.9	54.4 ± 10.2	43.7 ± 12.4
Age at onset (mean ± SD)	32.8 ± 10.9	29.5 ± 8.5	34.9 ± 11.8	43.4 ± 11.6	--
Disease duration (mean ± SD)	11.1 ± 7.9	10.5 ± 8.2	12.3 ± 9.3	11.0 ± 7.7	--
Expanded disability status scale	4.7 ± 2.2	2.9 ± 1.5	6.0 ± 1.3	6.8 ± 0.9	--
Progression index (EDSS/MS duration)	0.5 ± 0.4	0.3 ± 0.2	0.6 ± 0.5	0.7 ± 0.4	--

### Genotyping of *LINGO1* rs9652490 and rs18856808 polymorphisms

Genomic DNA was obtained from peripheral leukocytes and purified according to standard procedures. Two polymorphisms of *LINGO1* gene, rs9652490 A/G and rs11856808 C/T, were genotyped using KASPar assays according to the manufacturer’s protocol (http://www.kbioscience.co.uk). PCR was performed on a 96-well Tetrad 2 Peltier Thermal Cycler (BIO-RAD, Hercules, CA). PCR KASPar’s protocol was performed as following: a denaturation step of 10 min, twenty–eight cycles of 15 sec denaturing at 94°C, annealing of 20 sec at 57°C, and extension of 30 sec at 72°C. PCR were followed by a final extension step of 5 min at 72°C. Genotype calling was performed in an allelic discrimination analysis module of the 7300 High Throughput Sequence Detection System (ABI Sequence Detection Software v.1.2.3, Applied Biosystems, Foster City, CA, USA). We sequenced the SNP region in several individuals for each genotype for quality control of genotyping. Genotype success rate was 96.8%.

### Statistical analysis

Hardy-Weinberg equilibrium (HWE) was analyzed by the DeFinetti software (http://ihg.gsf.de/cgi-bin/hw/hwa1.pl). Allelic and genotype frequency analysis was performed with PLINK v.1.07 software (Shaun Purcell; http://pngu.mgh.harvard.edu/purcell/plink/). We performed Westfall and Young’s step-down max (T) permutation procedure implemented in PLINK v.1.07 by running 100.000 permutations to correct for multiple testing [29]. Level of statistical significance was considered at corrected p-values ≤ 0.05. The linkage disequilibrium between the two polymorphisms was calculated with Haploview Ver. 3. For categorical variables the intergroup comparison values were calculated by using the chi-square or Fisher's exact tests when appropriate. For continuous variables, the Kolmogorov-Smirnoff test was used to analyze normality in the distribution. Then, the Student two sample *t* test was used for variables that followed a normal distribution (age and age at onset), and the Mann–Whitney test was used for the duration of disease and the severity scores expanded disability status scale and progression index, because no normal distribution was observed for this parameter.The 95% confidence intervals were also calculated. The statistical power was calculated for the sample size of this study (this was determined from allele frequencies with a genetic model analyzing the frequency for carriers of the disease gene with OR = 1.5; p = 0.05) [30]. Bilateral and unilateral associations of the risk with the variant allele are as follows rs9652490 G = 80.1% and 87.7% and rs11856808 T = 91.9% and 96.7%, respectively. Haplotype reconstruction was performed using the program PHASE v2.1.1 [31]. We used the default model for recombination rate variation with 1000 iterations, 500 burn-in iterations and a thinning interval of 1 as described elsewhere [32].

## Results

The frequencies of *LINGO1* rs9652490 and rs11856808 genotypes and alleles in patients with MS did not differ from those of controls (Table 2). The genotype and allele frequencies in MS patients and healthy subjects were in Hardy-Weinberg’s equilibrium. Both SNPs were in linkage disequilibrium with LOD values equal to 48.67 and 36.17 among patients and controls subjects, respectively (r-squared = 0.46 and 0.48, respectively). Mean age at onset of MS did not differ significantly between patients carrying *LINGO1* rs9652490 A/A (mean ± SD = 33.0 ± 11.5 years), A/G (mean ± SD = 32.4 ± 9.8 years) and G/G (mean ± SD = 38.5 ± 24.7 years); (p = 0.818 for the comparison of carriers vs. non-carriers of variant alleles), and between patients with genotypes *LINGO1* rs11856808 C/C (mean ± SD = 32.5 ± 11.5 years), C/T (mean ± SD = 32.6 ± 10,6 years) and T/T (mean ± SD = 35.6 ± 10.9 years); (p = 0.835 for the comparison of carriers vs. non-carriers of variant alleles).

**Table 2:** ***LINGO1 *genotype and allelic variants of patients with multiple sclerosis (MS) and healthy volunteers**

	**MS PATIENTS (N = 293, 586 ALLELES)**	**CONTROLS (N = 318, 636 ALLELES)**	**Intergroup comparison values**	**MS WOMEN (N = 203, 406 ALLELES)**	**CONTROL WOMEN (N = 221, 442 ALLELES)**	**Intergroup comparison values**	**MS MEN (N = 90, 180 ALLELES)**	**CONTROL MEN (N = 97, 194 ALLELES)**	**Intergroup comparison values**
rs9652490 GENOTYPE A/A	197 (67.2%)	222 (69.8%)		139 (68.5%)	153 (69.2%)		58 (64.4%)	69 (71.1%)	
A/G	92 (31.4%)	86 (27.0%)	P = 0.197	62 (30.5%)	60 (27.1%)	P = 0.170	30 (33.3%)	26 (26.8%)	P = 0.651 *
G/G	4 (1.4%)	10 (3.1%)		2 (1.0%)	8 (3.6%)		2 (2.2%)	2 (2.1%)	
Allele A	486 (82.9%)	530 (83.3%)	--	340 (83.7%)	366 (82.8%)	--	146 (81.1%)	164 (84.5%)	--
Allele G	100 (17.1%)	106 (16.7%)	OR (95% CI)	66 (16.3%)	76 (17.2%)	OR (95% CI)	34 (18.9%)	30 (15.5%)	OR (95% CI)
			1.03 (0.76-1.39)			0.94 (0.65-1.34)			1.27 (0.74-2.18)
			P = 0.853			P = 0.715			P = 0.380
rs11856808 GENOTYPE C/C	137 (46.8%)	145 (45.6%)		95 (46.8%)	101 (45.7%)		42 (46.7%)	44 (45.4%	
C/T	126 (43.0%)	149 (46.9%)	P = 0.407	90 (44.3%)	103 (46.6%)	P = 0.852	36 (40.0%)	46 (47.4%)	P = 0.313
T/T	30 (10.2%)	24 (7.5%)		18 (8.9%)	17 (7.7%)		12 (13.3%)	7 (7.2%)	
Allele C	400 (68.3%)	439 (69.0%)	--	280 (69.0%)	305 (69.0%)	--	120 (66.7%)	134 (69.1%)	--
Allele T	186 (31.7%)	197 (31.0%)	OR (95% CI)	126 (31.0%)	137 (31.0%)	OR (95% CI)	60 (33.3%)	60 (30.9%)	OR (95% CI)
			1.04 (0.81-1.32)			1.00 (0.75-1.34)			1.08 (0.60-1.43)
			P = 0.773			P = 0.990			P = 0.725

P values correspond to 3x2 contingency tables (exact test). * Fisher’s exact test.

The distribution of rs9652490 and rs11856808 allelic and genotype frequencies were not influenced by gender (Table 2). The distribution of the *LINGO1* rs9652490 and rs11856808 genotype and allelic frequencies did not differ between each MS phenotype and controls (Table 3) or in the severity scores: expanded disability status scale or progression index. Haplotype analyses indicated that the commonest rs9652490-rs11856808 haplotype was AC (63.5% among patients and 69.2% among control individuals), followed by GT (19.4% and 16.6%, respectively) and AT (15.2% and 14.2%, respectively). These analyses did not reveal any differences in haplotype frequencies on comparing patients and control individuals (p > 0.05 for all comparisons). Haplotypes did not differ when subgroups of patients were compared according to gender, age at onset, MS phenotypes or severity scores (p > 0.05 for all comparisons).

**Table 3:** ***LINGO1 *genotypes and allelic variants in patients with MS, and relation with the evolutive type of MS**

	**RELAPSING-REMITTING MS (N = 159; 318 ALLELES)**	**Intergroup comparison values**	**SECONDARY PROGRESSIVE MS (N = 91; 182 ALLELES)**	**Intergroup comparison values**	**PRIMARY PROGRESSIVE MS (N = 43; 86 ALLELES)**	**Intergroup comparison values**	**CONTROLS (N = 318, 636 ALLELES)**
rs9652490 GENOTYPE							
A/A	102 (64.2%)		62 (68.1%)		33 (76.7%)		222 (69.8%)
A/G	56 (35.2%)	P = 0.055	26 (28.6%)	P =0.956	10 (23.3%)	P = 0.405	86 (27.0%)
G/G	1 (0.6%)		3 (3.3%)		0 (0.0%)		10 (3.1%)
Allele A	260 (81.8%)	--	150 (82.4%)	--	76 (88.4%)	--	530 (83.3%)
Allele G	58 (18.2%)	OR (95% CI)	32 (17.6%)	OR (95% CI)	10 (11.6%)	OR (95% CI)	106 (16.7%)
		1.12 (0.78-1.59)		1.07 (0.69-1.65)		0.66 (0.33-1.31)	
		P = 0.544		P = 0.771		P = 0.232	
rs11856808 GENOTYPE							
C/C	67 (42.1%)		43 (47.3%)		27 (62.8%)		145 (45.6%)
C/T	77 (48.4%)	P = 0.670	35 (38.5%)	P = 0.095	14 (32.6%)	P = 0.112	149 (46.9%)
T/T	15 (9.4%)		13 (14.3%)		2 (4.7%)		24 (7.5%)
Allele C	211 (66.4%)	--	121 (66.5%)	--	68 (79.1%)	--	439 (69.0%)
Allele T	107 (33.6%)	OR (95% CI)	61 (33.5%)	OR (95% CI)	18 (20.9%)	OR (95% CI)	197 (31.0%)
		1.13 (0.85-1.51)		1.12 (0.79-1.60)		0.59 (0.34-1.02)	
		P = 0.404		P = 0.515		P = 0.056	

P values correspond to 3x2 contingency tables (exact test).

## Discussion

The possible role of LINGO1 in the pathogenesis of MS makes it reasonable to analyse the possible relationship between *LINGO1* polymorphisms and the risk of MS. In the present study, we found no significant differences in allele genotypes, or haplotypes frequencies for the rs9652490 and rs11856808 polymorphisms when comparing patients with MS and healthy control subjects. Nor were these polymorphisms related with the age at onset of MS or with the evolutive type of MS. The findings obtained, though negative, are novel and represent an incremental advance in the knowledge of the clinical implications of the *LINGO1* gene polymorphism.

Some experimental data suggest a possible role of *LINGO1* in the pathogenesis of MS: (a) Nogo-A expression has been found to be enhanced in surviving oligodendrocytes, while NgR has been found to be up-regulated in reactive astrocytes and macrophages/microglia in chronic active demyelinating lesions of MS [33], (b) TROY has been found to be up-regulated, whereas LINGO1 expression has been found to be reduced, in MS brains [34], (c) *LINGO1* knockout mice have shown earlier onset of myelination of CNS axons than the wild-type, and greater resistance to the development of myelin oligodendrocyte glycoprotein (MOG)-induced experimental autoimmune encephalomyelitis (EAE) [35]; (d) Treatment with antibody antagonists against LINGO1 function induces functional recovery and increases integrity of axons in MOG-induced EAE [35], and promotes oligodendrocyte precursor cell differentiation and remyelination in different experimental models of demyelination and remyelination [36].

The *LINGO1* SNPs analyzed in this study have been studied as putative risk biomarkers for other movement disorders. A recent meta-analysis, which included 3,972 essential tremor (ET) patients and 20,714 controls for the LINGO1 rs9652490 polymorphism, and 2,076 ET patients and 18,792 controls for the rs11856808 polymorphism, concluded that the rs11856808 polymorphism was related with increased risk for both total and familial ET, whereas the rs9652490 polymorphism was related with increased risk for familial ET [21]. With regard to Parkinson’s disease, another recent meta-analysis including 5,541 patients and 5,647 controls for the rs9652490 polymorphism and 3,276 patients and 3,371 controls for the rs11856808 polymorphism concluded that these polymorphisms could not be considered as major risk factors for susceptibility to PD [22].

Several SNPs have been described within the *LINGO1* gene and in the 3’ flanking region (Figure 1). It is to be noted that most genetic association studies on *LINGO1* focused on the same SNPs which were analyzed in the present study [17-19,21-24,37-50]. Although several *LINGO1* nonsynonymous SNPs have been described, namely rs113329801, rs201732477, rs112205560, rs150289554, rs113096707, rs188738703, rs200688402, rs200463885, rs9855, rs201517725, rs184237450, rs77436810, rs193100227, rs111741384, rs202233236, rs199976207, rs199628078, rs201438433, rs140914739, rs200528664, rs111605415 and rs202223502, none of these SNPs show a minor allele frequency over 0.0005 (see the website http://www.ncbi.nlm.nih.gov/projects/SNP/snp_ref.cgi?showRare=on&chooseRs=all&go=Go&locusId=84894). Therefore, the identification of one heterozygous individual out of 1000 individuals studied is to be expected. This precluded the analysis of these nonsynonymous SNPs as putative risk factors. Nevertheless, the two SNPs studied are linked with other SNPs within the coding region of the *LINGO1* gene. Figure 1 show that linkage varies depending on the population studied, being low in individuals of African descent, high in individuals of Oriental descent or in related populations (of Amerindian descent), and intermediate in individuals of Caucasian descent. The Italian Tuscany population shows a higher linkage than other Caucasian individuals, probably due to genetic admixture with other Mediterranean populations. This admixture took also place in Spain. The Figure indicates that the two SNPs analyzed in this study are linked to the rest of the SNPs shown in the Figure.

The present study has some limitations. First, the size of analyzed cohorts may not be sufficient for strict conclusions about *LINGO1* role in MS. As was shown in a previous publication about the role of LINGO1 in essential tremor risk, individual studies of small number of patients gave very contradictory results [21]. Second, although the sample size is adequate to detect an OR as small as 1.5, a more modest association would not be detected. Third, because EDSS or progression index are not completely adequate measures of disease severity, the negative association observed in this study does not rule out a putative association with disease severity. Moreover, because the cohort study included MS patients with different degrees of severity, it is not adequate for the investigation of the influence of *LINGO1* genotypes on the disability or severity of MS (the ideal study for this purpose should include genotyping of patients with a recent diagnosis of MS with similar follow-up periods).

## Conclusions

In summary, taking in account the limitations of the present study, our results suggest that rs9652490 and rs11856808 genotype and allelic variants are not related with the risk for MS in Caucasian Spanish people.

## Competing interests

The authors declare that they have no competing interests.

## Authors’ contributions

EGM participated in the conception and design of the study, acquisition of data, analysis and interpretation of data, drafting of the manuscript, critical revision of the manuscript, administrative, technical, and material support, supervision, and obtaining funding. OLB participated in acquisition of data, analysis and interpretation of data, drafting of the manuscript, critical revision of the manuscript, administrative, technical, and material support. CM participated in acquisition of data, analysis and interpretation of data, critical revision of the manuscript, administrative, technical, and material support. PP participated in the conception and design of the study, acquisition of data, analysis and interpretation of data, drafting of the manuscript, critical revision of the manuscript, administrative, technical, and material support, supervision, and obtaining funding. JBL participated in acquisition of data, and critical revision of the manuscript. JM participated in acquisition of data, and critical revision of the manuscript. PC participated in acquisition of data, and critical revision of the manuscript. MDS participated in acquisition of data, and critical revision of the manuscript. DP participated in acquisition of data, and critical revision of the manuscript. LTF participated in acquisition of data, and critical revision of the manuscript. HAN participated in acquisition of data, analysis and interpretation of data, critical revision of the manuscript, administrative, technical, and material support. LAP participated in acquisition of data, and critical revision of the manuscript. DT participated in acquisition of data, and critical revision of the manuscript. EL participated in acquisition of data, and critical revision of the manuscript. JFPN participated in acquisition of data, and critical revision of the manuscript. JAGA participated in the conception and design of the study, acquisition of data, analysis and interpretation of data, drafting of the manuscript, critical revision of the manuscript, administrative, technical, and material support, supervision, and obtaining funding. FJJJ participated in the conception and design of the study, acquisition of data, analysis and interpretation of data, drafting of the manuscript, critical revision of the manuscript, administrative, technical, and material support, and supervision. All authors read and approved the final manuscript.

## Pre-publication history

The pre-publication history for this paper can be accessed here:

http://www.biomedcentral.com/1471-2377/13/34/prepub

## References

[B1] Giordano M D’Alfonso S Momigliano-Richiardi P Genetics of multiple sclerosis: linkage and association studies Am J Pharmacogenomics 2002 2 37 58 10.2165/00129785-200202010-00004 12083953

[B2] Dyment DA Ebers GC Sadovnick AD Genetics of multiple sclerosis Lancet Neurol 2004 3 104 110 10.1016/S1474-4422(03)00663-X 14747002

[B3] Ramagopalan SV Deluca GC Degenhardt A Ebers GC The genetics of clinical outcome in multiple sclerosis J Neuroimmunol 2008 201–202 183 199 18632165 10.1016/j.jneuroim.2008.02.016

[B4] Pugliatti M Harbo HF Holmoy T Kampman MT Myhr KM Riise T Wolfson C Environmental risk factors in multiple sclerosis Acta Neurol Scand Suppl 2008 188 34 40 18439219 10.1111/j.1600-0404.2008.01029.x

[B5] Duque B Sepulcre J Bejarano B Samaranch L Pastor P Villoslada P Memory decline evolves independently of disease activity in MS Mult Scler 2008 14 947 953 10.1177/1352458508089686 18573817

[B6] Baranzini SE Revealing the genetic basis of multiple sclerosis: are we there yet? Curr Opin Genet Dev 2011 21 317 324 10.1016/j.gde.2010.12.006 21247752 PMC3105160

[B7] Kemppinen A Sawcer S Compston A Genome-wide association studies in multiple sclerosis: lessons and future prospects Brief Funct Genomics 2011 10 61 70 10.1093/bfgp/elr004 21310812

[B8] Sawcer S Hellenthal G Pirinen M Spencer CC Patsopoulos NA Moutsianas L Dilthey A Su Z Freeman C Hunt SE Genetic risk and a primary role for cell-mediated immune mechanisms in multiple sclerosis Nature 2011 476 214 219 10.1038/nature10251 21833088 PMC3182531

[B9] Lassmann H Axonal injury in multiple sclerosis J Neurol Neurosurg Psychiatry 2003 74 695 697 10.1136/jnnp.74.6.695 12754330 PMC1738488

[B10] Domeniconi M Filbin MT Overcoming inhibitors in myelin to promote axonal regeneration J Neurol Sci 2005 233 43 47 10.1016/j.jns.2005.03.023 15949495

[B11] Mi S Miller RH Lee X Scott ML Shulag-Morskaya S Shao Z Chang J Thill G Levesque M Zhang M LINGO-1 negatively regulates myelination by oligodendrocytes Nat Neurosci 2005 8 745 751 10.1038/nn1460 15895088

[B12] Lee X Yang Z Shao Z Rosenberg SS Levesque M Pepinsky RB Qiu M Miller RH Chan JR Mi S NGF regulates the expression of axonal LINGO-1 to inhibit oligodendrocyte differentiation and myelination J Neurosci 2007 27 220 225 10.1523/JNEUROSCI.4175-06.2007 17202489 PMC6672289

[B13] Carim-Todd L Escarceller M Estivill X Sumoy L LRRN6A/LERN1 (leucine-rich repeat neuronal protein 1), a novel gene with enriched expression in limbic system and neocortex Eur J Neurosci 2003 18 3167 3182 10.1111/j.1460-9568.2003.03003.x 14686891

[B14] Mi S Lee X Shao Z Thill G Ji B Relton J Levesque M Allaire N Perrin S Sands B LINGO-1 is a component of the Nogo-66 receptor/p75 signaling complex Nat Neurosci 2004 7 221 228 10.1038/nn1188 14966521

[B15] Park JB Yiu G Kaneko S Wang J Chang J He XL Garcia KC He Z A TNF receptor family member, TROY, is a coreceptor with Nogo receptor in mediating the inhibitory activity of myelin inhibitors Neuron 2005 45 345 351 10.1016/j.neuron.2004.12.040 15694321

[B16] Shao Z Browning JL Lee X Scott ML Shulga-Morskaya S Allaire N Thill G Levesque M Sah D McCoy JM TAJ/TROY, an orphan TNF receptor family member, binds Nogo-66 receptor 1 and regulates axonal regeneration Neuron 2005 45 353 359 10.1016/j.neuron.2004.12.050 15694322

[B17] Stefansson H Steinberg S Petursson H Gustafsson O Gudjonsdottir IH Jonsdottir GA Palsson ST Jonsson T Saemundsdottir J Bjornsdottir G Variant in the sequence of the LINGO1 gene confers risk of essential tremor Nat Genet 2009 41 277 279 10.1038/ng.299 19182806 PMC3740956

[B18] Vilarino-Guell C Ross OA Wider C Jasinska-Myga B Cobb SA Soto-Ortolaza AI Kachergus JM Keeling BH Dachsel JC Melrose HL LINGO1 rs9652490 is associated with essential tremor and Parkinson disease Parkinsonism Relat Disord 2010 16 109 111 10.1016/j.parkreldis.2009.08.006 19720553 PMC2844122

[B19] Vilarino-Guell C Wider C Ross OA Jasinska-Myga B Kachergus J Cobb SA Soto-Ortolaza AI Behrouz B Heckman MG Diehl NN LINGO1 and LINGO2 variants are associated with essential tremor and Parkinson disease Neurogenetics 2010 11 401 408 10.1007/s10048-010-0241-x 20369371 PMC3930084

[B20] Deng H Gu S Jankovic J LINGO1 variants in essential tremor and Parkinson’s disease Acta Neurol Scand 2012 125 1 7 21470193 10.1111/j.1600-0404.2011.01516.x

[B21] Jimenez-Jimenez FJ Garcia-Martin E Lorenzo-Betancor O Pastor P Alonso-Navarro H Agundez JA LINGO1 and risk for essential tremor: results of a meta-analysis of rs9652490 and rs11856808 J Neurol Sci 2012 317 52 57 10.1016/j.jns.2012.02.030 22425540

[B22] Agundez JA Lorenzo-Betancor O Pastor P Garcia-Martin E Luengo A Alonso-Navarro H Jimenez-Jimenez FJ LINGO1 rs9652490 and rs11856808 are not associated with the risk of Parkinson’s disease: results of a meta-analysis Parkinsonism Relat Disord 2012 18 657 659 10.1016/j.parkreldis.2011.09.005 21955595

[B23] Lorenzo-Betancor O Samaranch L Garcia-Martin E Cervantes S Agundez JA Jimenez-Jimenez FJ Alonso-Navarro H Luengo A Coria F Lorenzo E LINGO1 gene analysis in Parkinson’s disease phenotypes Mov Disord 2011 26 722 727 10.1002/mds.23452 21506150

[B24] Lorenzo-Betancor O Garcia-Martin E Cervantes S Agundez JA Jimenez-Jimenez FJ Alonso-Navarro H Luengo A Coria F Lorenzo E Irigoyen J Pastor P Lack of association of LINGO1 rs9652490 and rs11856808 SNPs with familial essential tremor Eur J Neurol 2011 18 1085 1089 10.1111/j.1468-1331.2010.03251.x 21219542

[B25] McDonald WI Compston A Edan G Goodkin D Hartung HP Lublin FD McFarland HF Paty DW Polman CH Reingold SC Recommended diagnostic criteria for multiple sclerosis: guidelines from the international panel on the diagnosis of multiple sclerosis Ann Neurol 2001 50 121 127 10.1002/ana.1032 11456302

[B26] Martinez C Garcia-Martin E Benito-Leon J Calleja P Diaz-Sanchez M Pisa D Alonso-Navarro H Ayuso-Peralta L Torrecilla D Agundez JA Jimenez-Jimenez FJ Paraoxonase 1 polymorphisms are not related with the risk for multiple sclerosis Neuromolecular Med 2010 12 217 223 10.1007/s12017-009-8095-9 19826962

[B27] Garcia-Martin E Martinez C Benito-Leon J Calleja P Diaz-Sanchez M Pisa D Alonso-Navarro H Ayuso-Peralta L Torrecilla D Agundez JA Jimenez-Jimenez FJ Histamine-N-methyl transferase polymorphism and risk for multiple sclerosis Eur J Neurol 2010 17 335 338 10.1111/j.1468-1331.2009.02720.x 19538200

[B28] Agundez JA Arroyo R Ledesma MC Martinez C Ladero JM de Andres C Jimenez-Jimenez FJ Molina JA Alvarez-Cermeno JC Varela de Seijas E Frequency of CYP2D6 allelic variants in multiple sclerosis Acta Neurol Scand 1995 92 464 467 10.1111/j.1600-0447.1995.tb09614.x 8750111

[B29] Purcell S Neale B Todd-Brown K Thomas L Ferreira MA Bender D Maller J Sklar P de Bakker PI Daly MJ Sham PC PLINK: a tool set for whole-genome association and population-based linkage analyses Am J Hum Genet 2007 81 559 575 10.1086/519795 17701901 PMC1950838

[B30] Pértega Diaz S Pita Fernández S Cálculo del poder estadístico de un estudio Cadernos de atención primaria 2003 10 59 6323614145

[B31] Stephens M Donnelly P A comparison of bayesian methods for haplotype reconstruction from population genotype data Am J Hum Genet 2003 73 1162 1169 10.1086/379378 14574645 PMC1180495

[B32] Agundez JA Golka K Martinez C Selinski S Blaszkewicz M Garcia-Martin E Unraveling ambiguous NAT2 genotyping data Clin Chem 2008 54 1390 1394 10.1373/clinchem.2008.105569 18664443

[B33] Satoh J Onoue H Arima K Yamamura T Nogo-A and nogo receptor expression in demyelinating lesions of multiple sclerosis J Neuropathol Exp Neurol 2005 64 129 138 15751227 10.1093/jnen/64.2.129

[B34] Satoh J Tabunoki H Yamamura T Arima K Konno H TROY and LINGO-1 expression in astrocytes and macrophages/microglia in multiple sclerosis lesions Neuropathol Appl Neurobiol 2007 33 99 107 17239012 10.1111/j.1365-2990.2006.00787.x

[B35] Mi S Hu B Hahm K Luo Y Kam Hui ES Yuan Q Wong WM Wang L Su H Chu TH LINGO-1 antagonist promotes spinal cord remyelination and axonal integrity in MOG-induced experimental autoimmune encephalomyelitis Nat Med 2007 13 1228 1233 10.1038/nm1664 17906634

[B36] Mi S Miller RH Tang W Lee X Hu B Wu W Zhang Y Shields CB Miklasz S Shea D Promotion of central nervous system remyelination by induced differentiation of oligodendrocyte precursor cells Ann Neurol 2009 65 304 315 10.1002/ana.21581 19334062

[B37] Wu Y Wang X Xu W Liu W Fang F Ding J Song Y Chen S Genetic variation in LINGO-1 (rs9652490) and risk of Parkinson’s disease: twelve studies and a meta-analysis Neurosci Lett 2012 522 67 72 10.1016/j.neulet.2012.06.018 22710005

[B38] Annesi F De Marco EV Rocca FE Nicoletti A Pugliese P Nicoletti G Arabia G Tarantino P De Mari M Lamberti P Association study between the LINGO1 gene and Parkinson’s disease in the Italian population Parkinsonism Relat Disord 2011 17 638 641 10.1016/j.parkreldis.2011.06.020 21752692

[B39] Radovica I Inashkina I Smeltere L Vitols E Jankevics E Screening of 10 SNPs of LINGO1 gene in patients with essential tremor in the Latvian population Parkinsonism Relat Disord 2012 18 93 95 10.1016/j.parkreldis.2011.06.006 21741293

[B40] Bourassa CV Riviere JB Dion PA Bernard G Diab S Panisset M Chouinard S Dupre N Fournier H Raelson J LINGO1 variants in the French-Canadian population PLoS One 2011 6 e16254 10.1371/journal.pone.0016254 21264305 PMC3019170

[B41] Wu YW Rong TY Li HH Xiao Q Fei QZ Tan EK Ding JQ Chen SD Analysis of Lingo1 variant in sporadic and familial essential tremor among Asians Acta Neurol Scand 2011 124 264 268 10.1111/j.1600-0404.2010.01466.x 21158743

[B42] Wu YR Tan EK Chen CM Kumar PM Lee-Chen GJ Chen ST Genetic analysis of “leucine-rich repeat (LRR) and immunoglobulin (Ig) domain-containing, Nogo receptor-interacting protein-1 (LINGO1)” in two independent Chinese parkinson’s disease populations Am J Med Genet B Neuropsychiatr Genet 2011 156B 99 103 20957646 10.1002/ajmg.b.31124

[B43] Guo Y Jankovic J Song Z Yang H Zheng W Le W Tang X Deng X Yang Y Deng S LINGO1 rs9652490 variant in Parkinson disease patients Neurosci Lett 2011 487 174 176 10.1016/j.neulet.2010.10.016 20951767

[B44] Zuo X Jiang H Guo JF Yu RH Sun QY Hu L Wang L Yao LY Shen L Pan Q Screening for two SNPs of LINGO1 gene in patients with essential tremor or sporadic Parkinson’s disease in Chinese population Neurosci Lett 2010 481 69 72 10.1016/j.neulet.2010.06.041 20600614

[B45] Klebe S Thier S Lorenz D Nothnagel M Schreiber S Klein C Hagenah J Kasten M Berg D Srulijes K LINGO1 is not associated with Parkinson’s disease in German patients Am J Med Genet B Neuropsychiatr Genet 2010 153B 1173 1178 20468067 10.1002/ajmg.b.31085

[B46] Clark LN Park N Kisselev S Rios E Lee JH Louis ED Replication of the LINGO1 gene association with essential tremor in a North American population Eur J Hum Genet 2010 18 838 843 10.1038/ejhg.2010.27 20372186 PMC2987362

[B47] Thier S Lorenz D Nothnagel M Stevanin G Durr A Nebel A Schreiber S Kuhlenbaumer G Deuschl G Klebe S LINGO1 polymorphisms are associated with essential tremor in Europeans Mov Disord 2010 25 717 723 10.1002/mds.22887 20310002

[B48] Bialecka M Kurzawski M Tan EK Drozdzik M Analysis of LINGO1 (rs9652490) polymorphism in sporadic Parkinson’s disease in a Polish population, and a meta-analysis Neurosci Lett 2010 472 53 55 10.1016/j.neulet.2010.01.055 20117178

[B49] Haubenberger D Hotzy C Pirker W Katzenschlager R Brucke T Zimprich F Auff E Zimprich A Role of LINGO1 polymorphisms in Parkinson’s disease Mov Disord 2009 24 2404 2407 19908305 10.1002/mds.22768 PMC2798904

[B50] Tan EK Teo YY Prakash KM Li R Lim HQ Angeles D Tan LC Au WL Yih Y Zhao Y LINGO1 variant increases risk of familial essential tremor Neurology 2009 73 1161 1162 10.1212/WNL.0b013e3181bacfc9 19805735 PMC2890998

